# Symptomatic Unilateral Triceps Medial Head Snapping and Subluxating Ulnar Nerve without Neuropathy after Push-Ups: A Case Report

**DOI:** 10.5704/MOJ.2207.023

**Published:** 2022-07

**Authors:** WLB Lim, F Han

**Affiliations:** 1 Department of Family Medicine, National University Health System, Singapore; 2 Department Orthopaedic Surgery, Ng Teng Fong General Hospital, Singapore

**Keywords:** snapping elbow, ulnar nerve subluxation, medial head triceps snapping, surgical treatment

## Abstract

The coexistence of ulnar nerve subluxation and snapping medial head triceps is an uncommon occurrence. There have been few studies and case reports since it was first described in 1970. In this article, we present a case in which the condition occurred after a push-up. We analysed the pathoanatomy of the condition, and reviewed the literature regarding potential causes, typical presentations of the coexistence of both ulnar nerve subluxation and medial snapping triceps and describe our surgical technique in treatment. Elbow pain is very often under evaluated as many physicians may not be aware that elbow pain could be attributed to the coexistence of both ulnar nerve subluxation and medial snapping triceps. A thorough evaluation with physical examination and imaging are recommended. Early surgery with an appropriate rehabilitation programme may hasten recovery and return to sports in patients who continue to remain symptomatic following a trial of conservative therapy.

## Introduction

Snapping of the elbow remains an uncommon condition infrequently reported and poorly understood. Elbow snapping can occur laterally or medially, and it is associated with either an intra-articular or extra-articular pathology. Intra-articular snapping is usually due to hypertrophic synovial plicas, annular ligament pathology or lateral meniscus debris^1^ while extra-articular snapping is associated with subluxation of either the ulnar nerve and/or a medial head of the triceps tendon. This is of clinical significance because elbow pain is often under-evaluated as many physicians may not be aware that elbow pain could be attributed to dislocation of the medial head of triceps tendon or subluxation of the ulnar nerve during elbow flexion.

This report presents a patient with medial elbow pain after performing push-ups while on military training.

## Case Report

A 22-year-old male, enlisted in the army for full-time national service, started to develop a snapping sensation over the left elbow followed by intense pain immediately after performing push-ups. There was no associated weakness or numbness of the upper limb. The pain and snapping of the elbow were worsened with push-ups during the eccentric contraction of the triceps during elbow flexion and lowering of the body.

**Fig 1:**
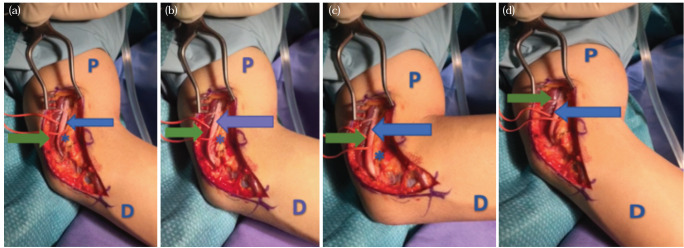
Subluxation of the Ulnar Nerve pre fixation during elbow flexion. (a) The elbow at 30° flexion. (b) The elbow at 70° flexion. (c) The elbow at 90° flexion. (d) Back to 30° elbow flexion. (Blue arrow: represent the ulnar nerve, Green arrow: the triceps medial head, and asterisks: the medial epicondyle. D: distal, P: proximal).

On examination, there was no localised bony tenderness at the elbow. Snapping of the triceps occurred with approximately 70° elbow flexion and a second snapping occasionally at approximately 100° after the ulnar nerve subluxation (Fig. 1). The elbow ligaments were stable with no ulnar nerve sensory or motor deficits. The range of motion of the elbow was full.

MRI showed medial subluxation of the medial head of triceps muscle and ulnar nerve, with focal nerve thickening and oedema over the medial epicondyle. There were no cartilaginous or bony abnormalities. The findings were suggestive of snapping triceps syndrome with ulnar nerve subluxation and ulnar neuropathy.

After unsuccessful non-operative management for more than three months, he underwent surgery with transposition of left ulnar nerve and resection of snapping medial head of triceps.

Through a medial approach to the elbow, the ulnar nerve and medial head of the triceps muscle were exposed. The Flexor Carpi Ulnaris (FCU) branches and Medial Antebrachial Cutaneous Nerve (MABC) were preserved. Intra-operatively, there was demonstrable subluxation of both the left ulnar nerve and medial triceps tendon on elbow flexion at about 70° and beyond 90°. consistent with the preoperative clinical and radiological findings.

**Fig 2:**
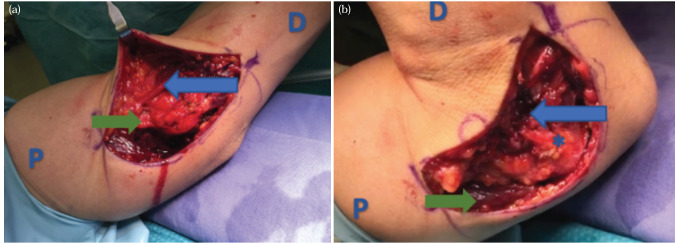
Transposition of the ulnar nerve performed using the submuscular technique. (a) The elbow at 20° flexion post fixation. (b) The elbow at 90° flexion post fixation. (Blue arrow: represent the ulnar nerve, Green arrow: the triceps medial head, and asterisks: the medial epicondyle. D: distal, P: proximal).

Decompression of the ulnar nerve from the arcade of Struthers distally to the two heads of the FCU was performed. The ulnar nerve was transposed using the submuscular technique, ensuring no kinking of the nerve and that the tunnel was free (Fig. 2). The elbow was moved through a range of -10° to 140°, ensuring that the ulnar nerve was free without kinking during the process.

Post-operative, the patient was allowed to range the elbow as tolerated. The sensation and motor function of the peripheral nerves of the left upper limb were intact.

The patient was progressively commenced on a rehabilitation programme and regular follow-up. The patient tolerated the rehabilitation programme without much difficulty and returned to army duty with full range of left elbow motion after three months. During the last follow-up at six months, he had no pain nor snapping at the medial aspect of the elbow, with full range of motion, and there was no tenderness.

## Discussion

Snapping of the elbow due to subluxation of the ulnar nerve and medial head triceps dislocation is uncommon. There have been several proposed causes of medial head dislocation during elbow flexion such as anatomical abnormalities, muscle hypertrophy, cubitus varus, osseous abnormalities or post-traumatic^2^ while the causes of ulnar nerve subluxation include congenital ligament laxity, dislocation of the medial head of the triceps or hypoplasia of the trochlea.

The present report describes medial elbow joint pain from snapping triceps and subluxation of the ulnar nerve in a young healthy male after doing push-ups. Usually with elbow flexion, the medial head of the triceps moves inward and displaces the ulnar nerve anteromedially by an average of 7.3mm^3^. The ulnar nerve typically subluxates at about 70° to 90° of elbow flexion causing the first snap, followed by the medial head of triceps dislocating at about 110° to 120° elbow flexion causing the second snap. The authors of this report postulate that injury occurs during the downward phase with eccentric loading of the triceps with the elbow bent. This is described as the angle of weakness because both the medial intermuscular septum that holds together the triceps muscle in position and the sheath that holds the ulnar nerve in the groove are prone to injury.

It has been reported that a significantly high percentage of patients with ulnar nerve subluxation did not get symptomatic relief after ulnar nerve transposition. A revision surgery had to be performed for persistent painful snapping of the elbow that was not identified before and during the index operation^4^. The authors recommend that a thorough evaluation of the presence of snapping triceps muscle be performed intra-operatively to ensure that there is no concurrent snapping triceps muscle pathology in the presence of ulnar nerve subluxation. In any uncertain cases, a dynamic ultrasound or MRI evaluation is required to establish the diagnosis in young patients who continue to remain symptomatic with elbow joint pain.

There have been several proposed separate surgical approaches for ulnar nerve subluxation or medial head triceps tendon dislocation but none for both ulnar nerve subluxation and medial head triceps tendon dislocation. For symptomatic recurrent ulnar nerve subluxation surgical techniques include anterior transposition or deep intramuscular implantation^5^. For symptomatic recurrent medial head triceps dislocation surgical techniques include lateral transposition of the dislocated medial portion of the triceps, partial resection of the dislocated triceps and medial epicondylectomy. Our patient failed a trial of conservative management after three months, after which he underwent surgical management of ulnar nerve transposition and partial resection of medial head of triceps. This allowed him to return to activities within six months. This management could be advocated in healthy active individuals who remained symptomatic after a period of conservative treatment

In conclusion, this report is to highlight a rare occurrence of ulnar nerve subluxation and medial head triceps dislocation sustained while performing push-ups. A thorough physical examination of the elbow joint is needed to identify any possible localised defects. In any doubtful cases, a dynamic ultrasound or MRI evaluation is useful to establish the diagnosis in young patients who continue to remain symptomatic with elbow joint pain. A thorough evaluation of the presence of snapping of the medial head triceps muscle has to be performed intra-operatively to ensure that there is no concurrent snapping triceps muscle pathology in the presence of ulnar nerve subluxation. If the patient continues to remain symptomatic despite a trial of conservative management, early surgery with an appropriate rehabilitation programme may hasten recovery and return the patient to sports and daily physical activities.
